# Culture-proven endophthalmitis associated with Boston Type I keratoprosthesis: clinical features, microbiologic aspects, and outcomes

**DOI:** 10.1186/s12348-025-00559-6

**Published:** 2026-05-21

**Authors:** Charles Zhang, Mariam Tadross, Michael M. Massengill, Guillermo Amescua, Victor L. Perez, Harry W. Flynn

**Affiliations:** 1 Department of Ophthalmology, Bascom Palmer Eye Institute, School of Medicine, University of Miami Miller, 900 NW 17th Street Miami, Miami, FL 33136 USA; 2 Miller School of Medicine, University of Miami, Miami, FL USA

**Keywords:** Keratoprosthesis endophthalmitis, Endophthalmitis, Boston keratoprosthesis

## Abstract

**Purpose:**

To characterize the clinical presentation, management, and outcomes of culture-proven endophthalmitis associated with the surgical use of a Boston Type I keratoprosthesis (Boston I KPro).

**Methods:**

A retrospective consecutive case series was conducted of culture-positive cases of endophthalmitis associated with the surgical use of Boston I KPro at a single tertiary referral center. Data were collected on patient demographics, transplant history, presenting features, management strategies, and outcomes.

**Results:**

Inclusion criteria were met by 9 eyes of 9 patients. The mean age was 76 ± 3 years. At presentation, all eyes were on chronic topical antibiotic prophylaxis. Initial vision was hand motion or worse in 8/9 (89%) of eyes. Presumed etiologies included early postoperative infection in 3/10 (30%), corneal melt with leaks in 3/10 (30%), corneal ulcer without leaks in 3/10 (30%). Vitreous cultures identified gram-positive organisms in 7/9 (78%), including *Staphylococcus aureus* in 3, *Streptococcus sp.* in 3, coagulative-negative *Staphylococcus* in 1, *Corynebacterium* in 1 and *Mycobacterium* in 1. Vitrectomy was performed in 8/9 (89%) of eyes with 3 eyes undergoing removal of the Boston I KPro. Despite prompt treatment, visual outcomes were poor with best-corrected visual acuity at last follow-up averaging 20/1600 with 2 eyes achieving ≥ 20/200 vision. Enucleation or evisceration was performed in 2 eyes.

**Conclusions:**

Culture-proven endophthalmitis after Boston I KPro implantation is a rare, devastating complication. Patients present rapidly with severe vision loss. Management often includes prompt vitrectomy. Visual prognosis is guarded with most eyes having worse than 20/200 vision at last follow-up.

## Introduction

The Boston Type I keratoprosthesis (Boston I KPro) is an artificial cornea designed to restore vision in patients with severe corneal disease who are poor candidates for traditional corneal transplantation [1]. Indications for Boston I KPro include eyes with multiple failed penetrating keratoplasties (PKP) or conditions associated with loss of the corneal limbal stem cells, such as autoimmune disease and chemical injury [2, 3]. The Boston I KPro has proven to be beneficial in challenging cases with approximately 45–89% of eyes achieving a best-corrected visual acuity (BCVA) of 20/200 or better [4]. With high device retention rates, ranging from 65% - 100% across various reports, many patients experience meaningful improvements in vision and quality of life [5, 6].

Despite these benefits, Boston I KPro implantation is associated with long-term complications [5]. Endophthalmitis is a devastating complication that has been reported in up to 12% of cases in one earlier series [7]. Boston I KPros are susceptible to infection because the conjunctiva and corneal graft tissue around the Boston I KPro can dissolve or melt, providing a direct route for surface flora into the eye [8]. Moreover, long-term use of topical antibiotics may alter the ocular surface microbiome and potentially encourage resistant organisms [9]. Nonetheless, advances in prophylactic regimens have significantly dropped the incidence of Boston I KPro-associated endophthalmitis down to 3–5% [6].

Given the relatively low incidence of Boston I KPro-associated endophthalmitis, there is limited high-quality evidence to guide management. No randomized trials have specifically addressed the optimal treatment approach and clinicians must extrapolate guidelines from small series. The current study aims to report the experience with culture-proven endophthalmitis associated with Boston I KPros at a single tertiary referral center. This study aims to focus on clinical presentation, microbiologic spectrum, treatment strategies, and clinical outcomes.

## Methods

The study was approved by the University of Miami Institutional Review Board and the research adhered to the tenets of the Declaration of Helsinki. All patients with a history of Boston I KPro-associated endophthalmitis seen at the University of Miami Bascom Palmer Eye Institute (BPEI) between January 1, 2013 and July 31, 2025 were reviewed. Cases were identified by querying institutional surgical and billing databases patients with Boston I KPros placed at BPEI or elsewhere that were diagnosed with endophthalmitis.

Inclusion criteria consisted of any eye with a history of Boston I Kpro surgery that subsequently developed infectious endophthalmitis with a positive vitreous culture either from the initial vitreous aspiration or subsequent pars plana vitrectomy (PPV). Eyes were excluded if the endophthalmitis was attributable to a source other than the Boston I KPro, such as those secondary to open-globe trauma or postoperative endophthalmitis from a different intraocular surgery. Eyes with culture-negative presumed endophthalmitis were not included in this series.

For each confirmed case, data were collected from the medical records in a standardized fashion. Patient demographics (age, sex), laterality of the affected eye, and details of the corneal transplant history were recorded. Additional ocular and medical history were reviewed. Visual acuity data was collected. Snellen acuity values were converted to logarithm of the minimum angle of resolution (logMAR) for analysis. For low vision notations such as counting fingers (CF, < 5/200 equivalent), hand motion (HM), light perception (LP), or no light perception (NLP), we assigned logMAR equivalents (approximately 2.1 for CF, 2.4 for HM, 2.7 for LP, and 3.0 for NLP) based on prior studies [10]. Microbiological data were obtained from laboratory records for all cases, including the results of vitreous cultures (from either needle tap or vitrectomy samples); all samples were grown on/in blood agar, chocolate agar, thioglycolate broth, Sabourand agar, Lowenstein-Jensen and Middlebrook agar. No attempts were made to culture the contact lenses or their storage solutions. Topical povidone-iodine (betadine) rinses were not used in any patients either preoperatively or postoperatively as part of the prophylactic regimen. Because of the limited sample size (*n* = 9), no comparative or correlation analyses were performed between infection severity and potential contributing factors such as virulence of the infecting organism, immunocompromised status, ocular surface condition, or aphakic state.

## Results

### Demographics and characterization of population of Boston type I K-Pro associated endophthalmitis

Boston I Kpro-associated endophthalmitis was identified in 9 eyes of 9 patients. A summary of the baseline patient and ocular characteristics is provided in Table 1. The mean age at the time of diagnosis of the infection was 76 ± 3 years (range 58–90 years), and 4/9 (44%) patients were male. The Boston I KPro placement was performed at BPEI in 8/9 (89%) of cases a mean of 339 ± 191 days (range 21 days to 4 years) prior. Of the 8 cases that had available operative reports, 6/8 (75%) had PPV at the time of Boston I KPro placement and 1/8 (13%) had a glaucoma drainage implant implanted at the time of Boston I KPro placement. The indications for Boston I KPro placement included a failure of PKP in 7/9 (78%) eyes, a longstanding Gunderson flap for neurotrophic keratopathy in 1 eye, and a history of Sarcoid cicatricial disease with prior failure of conjunctival limbal allograft, and simple limbal epithelial transplantation. In this series, the 7 eyes that had previously undergone PKP averaged 2.1 ± 0.4 PKPs per eye (range 1–4). The indication for the PKP included Fuch’s dystrophy in 2, pemphigoid in 1, keratoconus in 1, chemical burn in 1, corneal scar in 1, and neurotrophic cornea in 1. Previous glaucoma drainage implant surgery was performed on 7/9 (78%) of eyes. All eyes had prior cataract surgery; however, 5/9 (56%) were aphakic. Past medical history included diabetes mellitus in 4/9 (44%), and autoimmune disease in 3/9 (33%).

**Table 1:** Baseline demographics and ocular characteristics of patients with Boston Type I keratoprosthesis-associated endophthalmitis

Case	Gender	Age (years)	Previous BCVA	Previous Corneal/Conj Surgery	Indication for Corneal/Conj Surgery	KPro Surgery
**1**	Male	58	20/60	PKP x 4	Keratoconus	Type 1 KPro, PPV
**2**	Male	71	20/50	PKP x 3	Chemical Burn	Type 1 KPro
**3**	Female	86	20/40	PKP x 2	Corneal Scar	Type 1 KPro, PPV, GDI
**4**	Female	77	20/25	PKP, DSEAK	Fuch’s Dystrophy	Type 1 KPro, PPV
**5**	Female	65	CF	PKP	Neurotrophic Cornea	Type 1 KPro, PPV
**6**	Male	90	20/200	PKP, DSAEK	Fuch’s Dystrophy	Type 1 KPro*
**7**	Male	79	20/60	Gunderson Flap	Neurotrophic Cornea	Type 1 KPro, PPV
**8**	Female	80	20/150	PKP	Pemphigoid	Type 1 KPro
**9**	Female	75	20/800	KLAL, SLET	Sarcoid Conjunctivitis	Type 1 KPro, PPV

*Performed at outside institution

BCVA = best corrected visual acuity; CF = counting fingers; DSAEK = Descemet stripping automated endothelial keratoplasty; GDI = glaucoma drainage implant; KLAL = keratolimbal allograft; KPro = keratoprosthesis; PKP = penetrating keratoplasty; PPV = pars plana vitrectomy; SLET = simple limbal epithelial transplantation

At the last regular follow-up visit prior to infection, the mean BCVA was 0.8 ± 0.2 logMAR (approximately 20/125 Snellen equivalent, range 20/40 to CF), with 6/9 (67%) eyes seeing 20/200 or better. All patients were maintained on chronic topical antibiotic prophylaxis with either moxifloxacin or polymyxin B/trimethoprim, used one to four times daily in the eye with the KPro. Three patients received a secondary agent for prophylaxis, which included either topical gentamicin, tobramycin, or ciprofloxacin applied 1–4 times daily. Only 5/9 (56%) had a bandage contact in place at the time of diagnosis of endophthalmitis due to the remaining having a prior tarsorrhaphy. These contact lenses were washed regularly with povidone iodine.

### Presentations and management

**Fig. 1:**
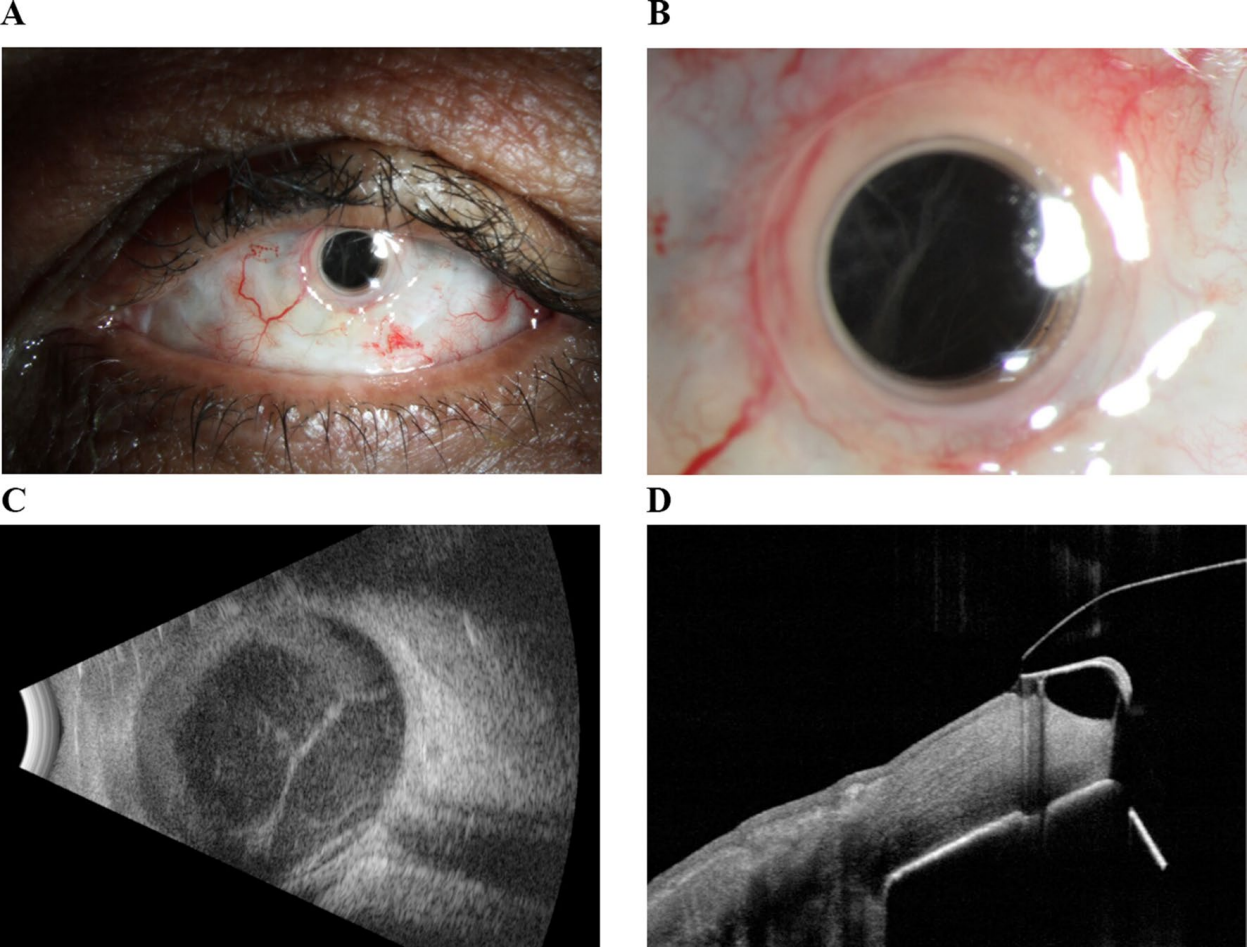
Representative imaging from patients with culture-proven Boston Type I Keratoprosthesis–associated endophthalmitis. **A** - Slit-lamp photograph of an eye presenting with acute vision loss due to *Staphylococcus aureus* infection. **B** - Magnified view from panel A demonstrating dense fibrin within the anterior chamber. **C** - B-scan ultrasound (longitudinal, 9-o’clock meridian) showing dense vitritis and vitreous membranes in an eye with *Streptococcus pyogenes*. **D** - Anterior segment optical coherence tomography revealing a gap at the graft–host interface in an eye infected with *Staphylococcus warneri*

During the 2013–2025 period, a total of 341 Boston I KPro surgeries were performed at BPEI. The occurrence of 9 instances of Boston I KPro-related endophthalmitis in this cohort corresponds to an incidence of approximately 2.6%. A summary of the presentations and management is provided in Table 2. At the time of diagnosis, the mean BCVA was logMAR 2.4 ± 0.2, (approximately HM Snellen equivalent, range 20/200 to LP) with 1/9 (11%) having 20/200 or better vision. On examination, all eyes had significant anterior segment (AC) inflammation (Fig. 1A and B). Clinically apparent vitritis was seen in 5/9 (56%) of eyes with 1 eye having a choroidal detachment (Fig. 1C). The presumed inciting causes for infection varied: 3/10 (30%) had endophthalmitis in the early postoperative period within 6 weeks of surgery, 3/10 (30%) had a significant corneal melt around the Boston I KPro with a positive Seidel test (Fig. 1D), and 3/10 (30%) had an infectious-appearing corneal infiltrate or ulcer without Seidel positivity.

**Table 2:** Ocular characteristics at presentation and interventions of patients with Boston Type I keratoprosthesis-associated endophthalmitis

Case	Time since KPro (days)	Presenting BCVA	Organism	Primary Treatment	Time to Vitrectomy	Surgical Intervention
**1**	27	LP	*Staphylococcus aureus*	Tap V/C	N/A	N/A
**2**	1460	HM	*Staphylococcus aureus*	Tap V/C	Next Day	PPV, V/C
**3**	890	20/200	*Mycobacterium*	Tap V/C	5 days	PPV, V/C/Vori
**4**	41	LP	*Streptococcus mitis*	Tap V/C/Vori	2 days	PPV, V/C/D
**5**	90	LP	*Staphylococcus aureus*	Tap V/C	Next Day	PPV, Removal of KPro, TPK, V/C/Vori
**6**	Unknown	HM	*Corynebacterium*	Tap V/C	Next Day	PPV, Removal of KPro, TPK, V/C/Vori
**7**	117	HM	*Streptococcus pyogenes*	Tap V/C	Next Day	PPV, V/C/D
**8**	21	LP	*Streptococcus pyogenes*	Tap V/C	3 days	PPV, Removal of KPro, Removal of IOL, TPK, V/C
**9**	64	HM	*Straphylococcus warneri*	Tap V/C	Next Day	PPV, V/C/D

BCVA = best corrected visual acuity; C = intravitreal Ceftazidime; D = intravitreal Dexamethasone; HM = hand motions; IOL = intraocular lens; KPro = keratoprosthesis; LP = light perception; PPV = pars plana vitrectomy; Tap = vitreous paracentesis and intravitreal injection; TPK = therapeutic penetrating keratoplasty; V = intravitreal Vancomycin; Vori = intravitreal Voriconazole

Initial therapy in every case included a vitreous aspiration for culture, immediately followed by intravitreal injection of vancomycin (1 mg/0.1 mL) and ceftazidime (2.25 mg/0.1 mL) in all 9 eyes with one patient receiving additional intravitreal injection of voriconazole. A prompt PPV for endophthalmitis within 24 h of presentation was performed in 5/9 (56%) of eyes and within 1 week in 3/4 (75%) of the remaining eyes. Among those undergoing PPV, the Boston I Boston I KPro was removed in 3/8 (38%) and 1 eye had explantation of the IOL.

Microbiological findings confirmed bacterial etiology in all cases, with gram-positive organisms being the most frequent culprits. Gram-positive bacteria grew in 7/9 (78%) isolates with *Staphylococcus aureus* in 3 eyes, *Streptococcus* in 3 and coagulase negative *Staphylococcus* (*S. warneri*) in 1. The remaining 2 isolates grew *Corynebacterium* and *Mycobacerium* species. Based on culture and susceptibility testing, four patients (44%) grew bacterial isolates (*Corynebacterium*,* Mycobacerium*, and *Staphylococcus aureus)* that were resistant to the specific set of topical antibiotics included in their prophylactic regimen. Notably, no fungi were isolated in any of the cases.

### Outcomes

All outcomes are summarized in Table 3. The mean duration of follow-up after the infection was 396 ± 90 days (approximately 13 months; range 36 days to 2.2 years). One eye underwent evisceration 21 days after initial diagnosis and another eye required an enucleation 38 days after diagnosis. Of the 3 eyes that had the Boston I KPro removed, 1 eye went on to require an additional PKP surgery 37 days later. A subsequent PPV was performed in 2/7 (28%) due to a tractional retinal detachment and persistent vitreous opacities.

**Table 3:** Summary of outcomes patients with Boston Type I keratoprosthesis-associated endophthalmitis

Case	Duration of Follow-Up (days)	BCVA at last follow-up	Additional Surgery
**1**	597	20/25	None
**2**	365	HM	None
**3**	90	N/A	Evisceration
**4**	180	20/200	PPV for TRD
**5**	405	LP	PKP
**6**	365	LP	None
**7**	36	LP	None
**8**	727	N/A	Enucleation
**9**	803	20/250	PPV for Vitreous Debris

BCVA = best corrected visual acuity; HM = hand motions; LP = light perception; PPV = pars plana vitrectomy; TRD = tractional retinal detachment

Visual outcomes were generally poor. Among the 7 patients with retained globes, the BCVA at last follow-up was logMAR 1.9 ± 0.4 (approximately 20/1600 Snellen equivalent, range 20/25 to LP). This represented a 11-line loss of visual acuity compared to baseline. Only 2/9 (22%) of eyes saw 20/200 or better at last follow up. 2 eyes in this series required evisceration or enucleation.

## Discussion

The current study demonstrates that Boston I KPro-associated endophthalmitis is uncommon. However, the visual outcomes can be devastating despite prompt management. The calculated incidence of endophthalmitis in the current study of 2.6% is consistent with improving trends reported in the literature. One systematic review reported the incidence of endophthalmitis to be 12% in the initial first decade of Boston I KPro use [11]. However, with the adoption of continuous topical antibiotic prophylaxis, the incidence decreased to 3% in the US, although this number included culture-negative cases [6]. All patients in the current study were maintained on continuous antibiotic prophylaxis with either moxifloxacin or polymyxin-B/trimethoprim. The microbiologic profile observed in the current study mirrors prior reports with similar prophylactic regimens and gram-positive bacteria accounting for a majority of isolates. Importantly, unlike endophthalmitis after routine cataract surgery where coagulase-negative *Staphylococcus* are the most common isolates, 6 of the 7 gram-positive isolates were due to *S. aureus* or *Streptococcus sp*., with only one case of coagulase-negative *Staphylococcus*. A plausible explanation is that the intensive prophylactic antibiotics in Boston I KPro patients may suppress many low-virulence skin flora. An earlier Boston I KPro series that did not use broad Gram-positive topical prophylaxis reported 70% of isolates being coagulase-negative *Staphylococcus* [12]. When infections do occur, they tend to involve organisms that are either more aggressive or more resistant. However, it is important to note that many patients undergoing Boston I Kpro tend to have abnormal ocular surface even prior to implantation.

An important finding is that despite the current study taking place in South Florida, no cases of culture-confirmed fungal endophthalmitis occurred in this series. This is surprising given that other centers have reported an emergence of fungal infections in Boston I KPro patients over the last decade [11, 13]. Various topical antibiotic prophylactic regimens have been described to reduce the risk of bacterial endophthalmitis, including fluoroquinolone-based monotherapy, polymyxin B/trimethoprim combinations, and vancomycin formulations. Vancomycin provides excellent coverage against Gram-positive organisms, including Staphylococcus aureus and Staphylococcus epidermidis, and has been associated with lower rates of bacterial infection in several studies [14, 15]. However, chronic use has also been linked to epithelial toxicity, poor epithelial healing, and a higher predisposition to fungal superinfection, particularly Candida species. This has led some authors to consider augmenting prophylaxis with topical amphotericin and 5% povidone iodine which was associated with a significant reduction in the rate of endophthalmitis in one study where 2/3s of isolates were fungal [16]. Due to limited data, it is unclear the optimal method of antibiotic prophylaxis to reduce the risk of endophthalmitis.

Management of Boston I KPro-associated endophthalmitis in this series involved prompt surgical intervention. All patients underwent a vitreous tap and intravitreal antibiotic injection at the time of diagnosis, and 89% underwent a PPV within 1 week with 33% undergoing concurrent explantation of the Boston I KPro. The decision to explant the keratoprosthesis was made in cases with severe corneal melt or where the prosthesis was unstable and suspected to harbor a biofilm that could perpetuate infection. Device removal is a difficult decision as it sacrifices the potential future vision that the Boston I KPro affords. This approach appears to have been beneficial in the current series as no cases of recurrent endophthalmitis were identified during a mean follow-up of 15 months. By comparison, a prior study performed early vitrectomy in 90% of eyes without Boston I KPro explantation and reported a 70% rate of recurrence of endophthalmitis a mean of 4 months later [12]. A separate small study also found that 1/3 eyes underwent Boston I KPro removal and similarly did not have recurrence of endophthalmitis [13]. 

Despite prompt management and no episodes of recurrence, outcomes were poor; 2 eyes underwent evisceration or enucleation and only 2 eyes recovered to ≥ 20/200 vision. This represents a profound decline from baseline vision which was ~ 20/125 on average. The globe loss rate is comparable to other series that reported a 17% incidence of evisceration in Boston I KPro eyes that developed endophthalmitis [17]. Such rates are markedly higher than those seen in endophthalmitis following routine cataract surgery, again pointing to the aggressive nature of infections in Boston I KPro eyes. It is worth highlighting that two patients achieved relatively favorable outcomes, regaining final acuities of 20/25 and 20/200. Interestingly, those two patients were among the ones who developed acute postoperative endophthalmitis after Boston I KPro implantation and had infections caused by *S. aureus* and *Streptococcus*. One possible explanation is that early postoperative infections might involve organisms that are less virulent and drug-resistant since the patients had a shorter duration of antibiotic exposure.

The current study has several limitations. It is a retrospective analysis of a relatively small number of cases at a single tertiary care center. The incidence of Boston I KPro-related endophthalmitis was estimated based on cases presenting to our institution, however, some patients received their Boston I KPro surgery elsewhere or may have sought care at outside facilities. Additionally, the current study cannot determine which interventions were most effective. Future studies pooling across multiple institutions are likely required to perform a more robust analysis of risk factors, management approaches, and outcomes.

## Conclusions

In summary, the current study reports that culture-proven Boston I KPro-associated endophthalmitis occurs in approximately 2.6% of cases which is in line with other modern studies. Patients typically present with acute pain and vision loss. Management often includes prompt vitrectomy and possible removal of the Boston I KPro device. Despite this, visual prognosis is guarded.

## Data Availability

All data generated or analyzed during this study are included in this published article.

## Abbreviations

Boston I KPro: Boston Type I keratoprosthesis
PKP: Penetrating keratoplasty
BCVA: Best corrected visual acuity
BPEI: University of Miami Bascom Palmer Eye Institute
PPV: Pars plana vitrectomy
logMAR: Logarithm of the minimum angle of resolution
CF: Counting fingers
HM: Hand motion
LP: Light perception
NLP: No light perception
AC: Anterior segment

## References

[1] Klufas MA, Colby KA (2010) The Boston keratoprosthesis. Int Ophthalmol Clin Summer 50(3):161–175. 10.1097/IIO.0b013e3181e20cca 10.1097/IIO.0b013e3181e20cca 20611026

[2] Harissi-Dagher M, Dohlman CH (2008) The Boston keratoprosthesis in severe ocular trauma. Can J Ophthalmol Apr 43(2):165–169. 10.3129/i08-009 10.3129/i08-009 18347618

[3] Ciralsky J, Papaliodis GN, Foster CS, Dohlman CH, Chodosh J (2010) Keratoprosthesis in autoimmune disease. Ocul Immunol Inflamm Aug 18(4):275–280. 10.3109/09273941003682300 10.3109/09273941003682300 20662659

[4] Ma JJ, Graney JM, Dohlman CH (2005) Repeat penetrating keratoplasty versus the Boston keratoprosthesis in graft failure. Int Ophthalmol Clin Fall 45(4):49–59. 10.1097/01.iio.0000176365.71016.28 10.1097/01.iio.0000176365.71016.28 16199966

[5] Lee WB, Shtein RM, Kaufman SC, Deng SX, Rosenblatt MI (2015) Boston keratoprosthesis: outcomes and complications: A report by the American academy of ophthalmology. Ophthalmology Jul 122(7):1504–1511. 10.1016/j.ophtha.2015.03.025 10.1016/j.ophtha.2015.03.025 25934510

[6] Ciolino JB, Belin MW, Todani A, Al-Arfaj K, Rudnisky CJ (2013) Boston keratoprosthesis type 1 study G. Retention of the Boston keratoprosthesis type 1: multicenter study results. Ophthalmology Jun 120(6):1195–1200. 10.1016/j.ophtha.2012.11.025 10.1016/j.ophtha.2012.11.025 PMC3674188 23499061

[7] Greiner MA, Li JY, Mannis MJ (2011) Longer-term vision outcomes and complications with the Boston type 1 keratoprosthesis at the university of California, Davis. Ophthalmology Aug 118(8):1543–1550. 10.1016/j.ophtha.2010.12.032 10.1016/j.ophtha.2010.12.032 21397948

[8] Aldave AJ, Kamal KM, Vo RC, Yu F (2009) The Boston type I keratoprosthesis: improving outcomes and expanding indications. Ophthalmology Apr 116(4):640–651. 10.1016/j.ophtha.2008.12.058 10.1016/j.ophtha.2008.12.058 19243830

[9] Bertino JS Jr (2009) Impact of antibiotic resistance in the management of ocular infections: the role of current and future antibiotics. Clin Ophthalmol 3:507–521. 10.2147/opth.s5778 19789660 10.2147/opth.s5778 PMC2754082

[10] Goldberg RA, Flynn HW Jr., Isom RF, Miller D, Gonzalez S (2012) An outbreak of Streptococcus endophthalmitis after intravitreal injection of bevacizumab. Am J Ophthalmol Feb 153(2):204–208e1. 10.1016/j.ajo.2011.11.035 10.1016/j.ajo.2011.11.035 PMC3266537 22264943

[11] Behlau I, Martin KV, Martin JN et al (2014) Infectious endophthalmitis in Boston keratoprosthesis: incidence and prevention. Acta Ophthalmol Nov 92(7):e546–e555. 10.1111/aos.12309 10.1111/aos.12309 24460594

[12] Ramchandran RS, Diloreto DA Jr., Chung MM et al (2012) Infectious endophthalmitis in adult eyes receiving Boston type I keratoprosthesis. Ophthalmology Apr 119(4):674–681. 10.1016/j.ophtha.2011.10.009 10.1016/j.ophtha.2011.10.009 22266108

[13] Chan CC, Holland EJ (2012) Infectious endophthalmitis after Boston type 1 keratoprosthesis implantation. Cornea Apr 31(4):346–349. 10.1097/ICO.0b013e31821eea2f 10.1097/ICO.0b013e31821eea2f 22314825

[14] Durand ML, Dohlman CH (2009) Successful prevention of bacterial endophthalmitis in eyes with the Boston keratoprosthesis. Cornea Sep 28(8):896–901. 10.1097/ICO.0b013e3181983982 10.1097/ICO.0b013e3181983982 19654525

[15] Yu JF, Huang YF (2012) Characteristics of endophthalmitis with Boston keratoprosthesis. Cornea May 31(5):594. 10.1097/ICO.0b013e3181fb865f 10.1097/ICO.0b013e3181fb865f 21968604

[16] Prabhasawat P, Chotikavanich S, Ngowyutagon P, Pinitpuwadol W (2021) Long-term outcomes of Boston type I Keratoprosthesis, and efficacy of amphotericin B and Povidone-Iodine in infection prophylaxis. Am J Ophthalmol Dec 232:40–48. 10.1016/j.ajo.2021.05.022 10.1016/j.ajo.2021.05.022 34102154

[17] Bostan C, Nayman T, Szigiato AA, Morfeq H, Harissi-Dagher M (2021) Endophthalmitis in eyes with the Boston type I keratoprosthesis: Incidence, Recurrence, risk Factors, and outcomes. Cornea Oct 1(10):1258–1266. 10.1097/ICO.000000000000264 10.1097/ICO.0000000000002641 33394754

